# Alternative splicing of exon 10 in the *tau *gene as a target for treatment of tauopathies

**DOI:** 10.1186/1471-2202-9-S2-S10

**Published:** 2008-12-03

**Authors:** Jianhua Zhou, Qingming Yu, Tie Zou

**Affiliations:** 1 Department of Medicine, Program in Neuroscience, University of Massachusetts Medical School, 364 Plantation Street, Worcester, MA 01605, USA

## Abstract

Tau aggregation is one of the major features in Alzheimer's disease and in several other tauopathies, including frontotemporal dementia with Parkinsonism linked to chromosome 17 (FTDP-17), and progressive supranuclear palsy (PSP). More than 35 mutations in the *tau *gene have been identified from FTDP-17 patients. A group of these mutations alters splicing of exon 10, resulting in an increase in exon 10 inclusion into *tau *mRNA. Abnormal splicing with inclusion of exon 10 into *tau *mRNA has also been observed in PSP and AD patients. These results indicate that abnormal splicing of exon 10, leading to the production of *tau *with exon 10, is probably one of the mechanisms by which tau accumulates and aggregates in tauopathic brains. Therefore, modulation of exon 10 splicing in the *tau *gene could potentially be targeted to prevent tauopathies. To identify small molecules or compounds that could potentially be developed into drugs to treat tauopathies, we established a cell-based high-throughput screening assay. In this review, we will discuss how realistic, specific biological molecules can be found to regulate exon 10 splicing in the *tau *gene for potential treatment of tauopathies.

## Alternative splicing of exon 10 in the *tau *gene

Splicing of pre-mRNA is a complicated process that is regulated by intron and exon sequences, SR (serine/arginine-rich) proteins, and other non-SR splicing factors, for example, heterogeneous nuclear ribonucleoproteins (hnRNPs). Alternative splicing occurs when the introns of a certain pre-mRNA are excised in more than one way, producing structurally and functionally different proteins from the same gene in different cells, or different developmental stages. It is estimated that more than 60% of human genes undergo alternative splicing, making the cellular process an essential mechanism for generating protein diversity [1-4]. Both constitutive and alternative splicing play important roles in the regulation of gene expression in eukaryotes [5]. A growing number of genetic diseases have been found to be caused by alternative or aberrant splicing events [6-9]. Mutations in *cis*-elements for splicing are primarily responsible for these diseases. However, tissue-specific splicing factors, such as those identified in amyotrophic lateral sclerosis (ALS), are also suggested to cause the aberrant pathogenic processing of pre-mRNA [10-13]. In addition, polymorphisms and genetic background contribute to variation in alternative splicing in specific genes, leading to modification of phenotypes in diseases (reviewed by Wang and Cooper [14]).

A typical exon is defined by its sequence, which is usually less than 300 base pairs long and, together with its surrounding introns, contains at least three core *cis*-elements: a 5' splicing site, a 3' splicing site, and a branch point [5,15]. The splicing events begin with recognition of these elements by assembly of the spliceosome, a complex consisting of different small nuclear ribonuclear particles (snRNPs; U1, U2AF65, U4/U6, and U5), SR proteins (SRP30C, SRP20, SRP40, SRP55, SC35, SF2, hTra2b1), and other factors, including SF1 [5] (reviewed by Kramer [15]). The 5' site is initially paired with U1 snRNP and then replaced by U6 snRNP. The large subunit of splicing factor U2AF binds to the polypyrimidine tract while SF1 interacts with the branch site [16]. SR proteins, on the other hand, function as trans-splicing factors that facilitate the interaction of U1 snRNP with the 5' splice site, and the assembly of spliceosomes. through interactions with U2AF [17], SF1, and other splicing factors. After a series of conformational changes, introns are enzymatically removed and exons are ligated at 5' and 3' splicing sites. Exons, as well as introns, contain *cis*-regulatory splicing elements that activate or inhibit the splicing of a specific exon. These elements include splicing enhancers and splicing silencers [18-21]. Due to the involvement of many factors in the process of alternative splicing, one would expect that any change of these elements in the overall equation would tip the balance of exon inclusion/exclusion.

**Figure 1:**
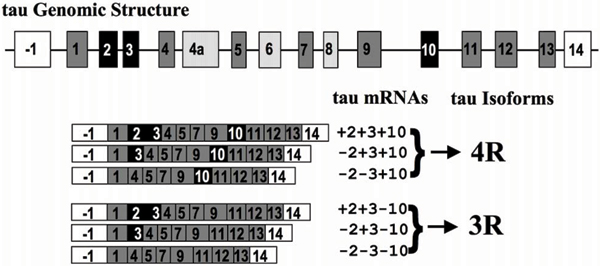
The *tau *genomic structure and splicing products. The *tau *gene has 16 exons. Exons 4A, 6, and 8 are not expressed in the human brain. Exons 2, 3, and 10 are alternatively spliced, producing six different mRNAs. The mRNA with or without exon 10 encodes a tau protein with four repeats (4R) or three repeats (3R), respectively. Adapted from Lee *et al*. [28].

The human *tau *gene is more than 100 kilobases long and has been mapped to chromosome 17q21. It contains 16 exons, with the first exon (-1) as a part of the promoter (Figure 1). Exons 4A, 6, and 8 are not present in the *tau *mRNAs in human brain [22]. A full-length tau protein is encoded by exons 1–13 and consists of 441 amino acids. Three exons (exons 2, 3, and 10) undergo alternative splicing, giving rise to six isoforms of tau (Figure 1). The fetal tau isoform excludes exons 2, 3, and 10, while adults express all six tau isoforms, which range from 352–441 amino acids in length, depending on inclusion or exclusion of exons 2, 3, and 10 [23]. One of the major characteristics of the tau protein is the presence of four carboxy-terminal tandem repeat sequences (of 31 or 32 amino acids), each of which is encoded by exons 9, 10, 11, and 12 [24-27]. Since exon 10, which encodes the second repeat, is alternatively spliced., isoforms of tau are classified as either 3R taus (three repeats) or 4R taus (four repeats) (Figure 1) [28].

Alternative splicing of exon 10 is developmentally regulated. It has been shown that >95% of *tau *mRNA in fetal brain excludes exon 10, while 40–50% of *tau *mRNAs in adult central nervous system includes this exon [29]. Exon 10 is relatively small at 93 base pairs. Exonic splicing enhancers, as well as an exonic splicing silencer, have been identified within this exon [29-31]. By analyzing mini-genes constructed from exon 9, intron 9, exon 10, intron 10 and exon 11 of the *tau *gene, we and others have also shown that additional *cis*-elements, including exon 9, exon 11, and trans-factors – for example, SRp20, SRp55, SRp75, SRp30c, ASF, SC35 and hTra2β1 – regulate exon 10 splicing [29,32-34]. Studies of *tau *pre-mRNA identified a stem-loop structure at the exon 10-intron 10 boundary, the disruption of which alters exon 10 splicing [30,35,36]. Several splicing factors, including hTra2β1, SF2/ASF, SRp55, and SRp30C, affect exon 10 splicing by directly binding to exonic enhancers [37,38]. However, it is unclear how other *cis*-elements and *trans*-factors are involved in exon 10 splicing.

Tau is abundantly expressed in the central nervous system, predominantly located in axons, and expression is also detectable in the axons of peripheral neurons. Only low levels of expression are observed in central nervous system astrocytes and oligodendrocytes [39-41]. The main function of tau is to bind and stabilize microtubules. The binding of tau to microtubules promotes their polymerization [39,42]. Analysis of the tau sequence revealed that tau has four microtubule-binding motifs that are located in four repeat regions at the carboxyl terminus of the protein within conserved 18 residue long binding elements separated by less conserved spacers of 13–14 amino acids [28]. 4R-tau isoforms, which contain an additional binding element, have a greater affinity for microtubules and are more efficient in promoting their polymerization than 3R-tau isoforms [43,44]. It is interesting that in adult human brain, the ratio of 3R-tau to 4R-tau isoforms is about 1 [43]. Therefore, one would expect that in a normal individual the ratio of 4R-tau/3R-tau proteins is balanced.

## Tauopathies and mutations in *Tau*

Tauopathies comprise a group of neurodegenerative disorders that share a common pathological feature: the formation of insoluble intraneuronal aggregates composed of filamentous hyperphosphorylated tau proteins [28,45]. Paired helical filaments and the hyperphosphorylation of tau are two of the most recognized molecular characteristics in Alzheimer's disease (AD) and several other neurodegenerative tauopathies, for example, frontotemporal dementia with Parkinsonism linked to chromosome 17 (FTDP-17) and progressive supranuclear palsy (PSP) [28]. The genetic relevance between *tau *and tauopathies came from linkage analysis of FTDP-17 and other neurodegenerative diseases, many of which were mapped to the region on chromosome 17q21–22 where the *tau *gene is located [46-50]. Subsequently, mutations were identified in the *tau *gene from FTDP-17 patients [48,51-53]. To date, at least 35 distinct pathogenic mutations in the *tau *gene that lead to the formation of filaments made of hyperphosphorylated tau [54] have been described in a large number of families with FTDP-17 (Table 1; ) [22,55]. Several other mutations have also been described in PSP, Pick's disease, and Parkinson's disease (Table 2; ) [56-60]. It has also been demonstrated that the majority of missense mutations that reside in coding regions of *tau *exons 9, 10, 12, or 13 alter the ability of tau to interact with microtubules and to promote microtubule assembly [51,52,59,61-63]. On the other hand, additional mutations (for example, three silent mutations in exon 10, a deletion mutation, seven substitutions of the intron following exon 10, and one mutation in intron 9) disrupt exon 10 splicing *cis*-elements (Table 1). This mostly results in the increased production of *tau *mRNA with exon 10 inclusion, shifting the 3R-tau/4R-tau ratio in favor of more 4R-tau [51,64-66]. It has been also demonstrated that the exonic mutations S305N, N279K, Δ280K, and N296H may not only alter tau functions, but also affect splicing enhancers, or destabilize the stem loop structure of *tau *pre-mRNA, causing abnormal exon 10 inclusion/exclusion [30,67]. All these results suggest that aberrant exon 10 splicing is one of the most important mechanisms in the pathogenesis of tauopathies.

**Table 1:** Mutations in the tau gene that are identified in FTDP-17 patients

**Exon or intron**	Mutations
EX1	Arg5His
EX9	Ile260Val; Leu266Val; Gly272Val
EX10	Asn279Lys; ΔK280; ΔK281; Asn296His; Pro301Thr; Pro301Ser; Pro301Leu; Gly303Val; Ser305Asn
EX11	Leu315Arg; Lys317Met; Ser320Phe
EX12	Gly335Ser; Gly335Val;Gln336Arg; Val363Ile; Val337Met; Glu342Val; Ser352Leu; Lys369Ile
EX13	Arg406Trp; Thr427Met
	
**IVS9**	**-10G>T**
**EX10**	**Asn279Lys**; **ΔK280; Asn296; Leu284; Ser305, Asn296His; Pro301Thr**
**IVS10**	**+3G>A; +11T>C; +12C>T; +13A>G; +14C>T; +16C>T; +19C>G**

Data are derived from . Mutations in bold affect exon 10 splicing. IVS, intervening sequence.

**Table 2:** Mutations in the tau gene that are identified in other tauopathies

Exon	Mutation	Disease
EX1	Arg5Leu	PSP
EX9	Lys257Thr	Pick
EX10	ΔN296	PSP
EX13	Gly389Arg (G>A mutation)	Pick
EX13	Gly389Arg (G>C mutation)	Pick
EX13	Gln424Lys	Parkinson's disease

Data are derived from . PSP, progressive supranuclear palsy.

In addition to the mutations that have been identified in FTDP-17 patients, it has been suggested that additional pathogenic mutations or polymorphisms in introns that affect exon 10 splicing may be present in tauopathies [28]. Analysis of tau protein and mRNA in PSP indicates that more 4R-tau is produced in these patients [68]. However, unlike other tauopathies, PSP has usually been considered to be sporadic in nature. Conrad *et al*. [69], however, demonstrated an association between a polymorphic dinucleotide repeat marker, (GT)n, found in intron 9 of the *tau *gene and PSP. These results were subsequently confirmed by several other groups [70,71]. Further studies indicate that a series of polymorphisms within the *tau *gene (for example, eight single nucleotide polymorphisms, one deletion, and the (TG)n repeat) are inherited in completed linkage disequilibrium, defining the *tau *gene as two extended haplotypes, H1 and H2 [72] (reviewed by Schraen-Maschke *et al. *[22]). Importantly, it was found that the most common allele (H1 haplotype) and genotype (H1/H1) were over-represented in PSP patients compared with normal controls. However, although the dinucleotide polymorphism is associated with extended H1/H2 haplotypes in disequilibrium, it has been considered a variation that may not have biological significance in the disease process. Taken together with the discoveries of the over-production of 4R-tau, and disequilibrium linkage to the *tau *gene in PSP patients, it is reasonable to presume that variations in intron 9 or intron 10 could be critical to influencing exon 10 inclusion/exclusion in these patients [71,73-75].

In contrast, dysregulation of *tau *pre-mRNA splicing contributing to AD pathogenesis is still under debate. Several groups have shown by quantitative RT-PCR that there is no direct correlation between the 4R/3R ratio and tau pathology in AD patients [73-75]. On the other hand, Glatz *et al*. [76] demonstrated that in the temporal cortex of many AD brains, production of 4R-tau is significantly elevated, suggesting the importance of exon 10 inclusion/exclusion in the pathogenesis of AD. Nevertheless, although mutations have only been identified in the *tau *gene from FTDP-17 patients, *tau *polymorphisms, or other factors that may affect *tau *splicing, appear to be genetic risk factors for neurodegenerative diseases with tauopathy, in particular in PSP and AD patients.

## Pharmacological intervention of *tau *splicing in tauopathy

Tau is hyperphosphorylated and forms aggregates in brains of tauopathic patients. One would expect that small molecules and drugs that prevent these processes could be identified and used as therapeutic treatments. Not surprisingly, extensive efforts have been made to identify these molecules to inhibit formation of tau filaments or tau hyperphosphorylation as the mainstream objective in the search for drugs for treatment of tauopathies [55,77]. However, the overlooked exon 10 splicing of the *tau *gene has been gaining ground during the past few years as an area of study for potential treatment of tauopathies.

Several well-documented technologies that target aberrant splicing of specific genes in the treatment of diseases have been discussed in a recent review by Gallo *et al. *[55]. Among them, antisense oligoribonucleotides have been most commonly cited. For instance, Kalbfuss *et al. *[78] demonstrated that oligoribonucleotides binding to E10 splicing junctions could suppress the predominant inclusion of exon 10 in *tau *mRNA in rat PC12 cells. Other examples of modulating alternative splicing with antisense oligoribonucleotides include the genes *SMN *and *Bcl-x *[21,79]. Another strategy that is often employed to stimulate the inclusion or exclusion of a specific exon is to use bifunctional oligoribonucleotides. These molecules are made of an antisense sequence designed to bind to an exonic element and a *trans*-factor sequence or peptide domain mimicking a *trans*-splicing factor [80-82]. Relative to antisense oligonuribonucleotides or bifunctional oligoribonucleotides, spliceosome-mediated RNA trans-splicing (SMaRT) is new. SMaRT is a technology that was developed based on the natural occurring *trans*-splicing event between two independently transcribed pre-mRNAs [83]. By taking advantage of this finding, Puttaraju *et al*. [83] developed a series of RNA pre-*trans*-splicing molecules capable of base-pairing to and *trans*-splicing with a conventional target pre-mRNA. They demonstrated that splicing of the conventional pre-mRNA was modified by these RNA pre-*trans*-splicing molecules both *in vitro *and in transfected cells. SMaRT has been tested in *tau *and *SMN *and shown great promise in correcting abnormal splicing of these two genes [83-85].

However, one specific area Gallo *et al*. [55] did not discuss in their review is whether small molecules or chemical compounds can potentially be used to treat diseases by targeting splicing defects. To provide proof-of-principle, we used neurodegenerative spinal muscular atrophy (SMA) as a model. SMA is caused by the homozygous loss of the *SMN1 *gene. *SMN2*, which differs from *SMN1 *by a single nucleotide within exon 7 that affects the efficiency of its incorporation into the mRNA transcript, is preserved in SMA patients [86,87]. Increased exon 7 insertion into *SMN2 *mRNA has been suggested as a potentially effective approach for SMA treatment [88]. Using a cell based assay we have developed, we identified the phosphatase inhibitor sodium orthovanadate in a small screen of chemicals known to affect signaling pathways [88]. Additional screening of a library of compounds identified aclarubicin, a chemotherapeutic anthracycline drug that is related to adriamycin [89], and indoprofen, a common non-steroidal anti-inflammatory drug (NSAID) [90]. Aclarubicin, but not adriamycin, increased the amount of full-length transcript produced by the *SMN2 *gene, which in turn restored SMN protein levels in fibroblasts isolated from a SMA patient. In addition, we showed that aclarubicin has no effects on the splicing of *Bcl-x*, *tau *and Myosin V genes [89], indicating a relatively specific effect of aclarubicin on the splicing of the *SMN2 *gene [89]. We conclude from our studies of *SMN *that relative specific small molecules able to reverse abnormal splicing may be identified for disease treatment.

## Conclusion

**Figure 2:**
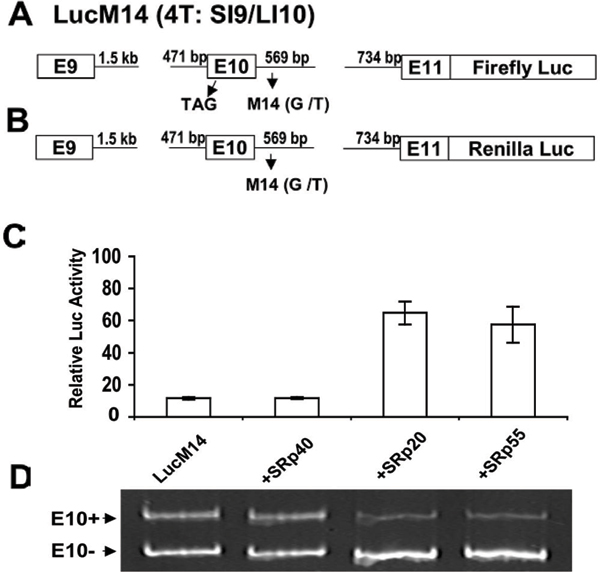
Validation of a cell-based luciferase system for measurement of exon 10 splicing in the *tau *gene (adapted from Yu *et al. *[91]). **(A) **The luciferase mini-gene construct. The firefly luciferase gene was fused downstream of exon 11 in frame with a SI9/LI10 mini-gene containing the M14 (G to T) mutation. A stop codon was introduced into exon 10 by mutagenizing position 73 in exon 10 (A to T). The nucleotide A of the initiation codon ATG in the luciferase gene was converted to T. **(B) **A control mini-gene with a fused *Renilla *luciferase to exon 11. **(C) **The firefly luciferase mini-gene (A) was co-transfected into SKN-MC cells with constructs of SRp40, SRp20, or SRp55. Relative firefly luciferase activities were normalized using the *Renilla *luciferase control (B). **(D) **RT-PCR from cells described in (C) was carried out to quantify exon 10 inclusion/exclusion.

Aberrant inclusion of exon 10 into *tau *mRNA promotes the increased production of 4R-tau and has been increasingly recognized as one of the causes of tauopathies in FTDP-17, PSP, and many AD patients [51,64-66,76]. The similarity between exon 7 splicing in the *SMN *gene and exon 10 splicing in the *tau *gene prompted us to design a cell-based assay using luciferase as a reporter for the identification of small molecules that can reverse abnormal exon 10 inclusion. The mini-reported genes are displayed in Figure 2[91]. When exon 10 is excluded, the luciferase is expressed, while inclusion of exon 10 shifts luciferase out of the reading frame, resulting in no luciferase activity. Compounds that stimulate luciferase activity would likely act by reducing exon 10 inclusion in the *tau *mRNA. Based on this assay, we have carried out low- and high-throughput screening. While characterizing more than 500 compounds that stimulated luciferase activity (T Zou *et al*., unpublished data), we speculate that it is not out of reach that relative specific small molecules will be identified for further development of drugs for treatment of tauopathies.

## List of abbreviations used

AD: Alzheimer's disease; ALS: amyotrophic lateral sclerosis; FTDP-17: frontotemporal dementia with Parkinsonism linked to chromosome 17; PSP: progressive supranuclear palsy; SMA: spinal muscular atrophy; SmaRT: spliceosome-mediated RNA trans-splicing; snRNP: small nuclear ribonuclear particle.

## Competing interests

The authors declare that they have no competing interests.
